# *Nucleospora hippocampi* n. sp., an Intranuclear Microsporidian Infecting the Seahorse *Hippocampus erectus* From China

**DOI:** 10.3389/fcimb.2022.882843

**Published:** 2022-05-04

**Authors:** Yuan Wang, Na Ying, Yanqing Huang, Xiong Zou, Xin Liu, Letian Li, Junfang Zhou, Shu Zhao, Rongrong Ma, Xincang Li, Hongxin Tan, Wenhong Fang

**Affiliations:** ^1^ East China Sea Fisheries Research Institute, China Academy of Fishery Sciences, Shanghai, China; ^2^ College of Fisheries and Life Science, Shanghai Ocean University, Shanghai, China; ^3^ College of Marine Sciences, Ningbo University, Ningbo, China

**Keywords:** seahorse, *Nucleospora*, transmission electron microscopy, intranuclear parasitism, intestinal disease

## Abstract

The life cycle, ultrastructure, and molecular phylogeny of a new intranuclear microsporidian, *Nucleospora hippocampi* n. sp., infecting the intestine of the *Hippocampus erectus*, were described. The histopathology revealed an extensive infection, mainly in the columnar epithelium of the intestinal mucosa layer. The enterocytes were the important target cell for *Nucleospora hippocampi* n. sp. infection. Transmission electron microscopy results showed that this microsporidian developed directly within the host cell nucleoplasm. In the intranuclear life cycle, the transformation from meront to sporogonial plasmodium was recognized by forming electron-dense disc structures, which were considered the polar tube precursors. The microsporidian showed the typical morphological characteristics of the family Enterocytozoonidae in the formation and development of spore organelles prior to the division of the sporogonial plasmodium. According to wet smear observation, eight spores were generally formed in a single host nucleus. Mature spores were elongated ovoids that were slightly bent and measured 1.93 × 0.97 μm. The isofilar polar tube was arranged in 7~8 coils in one row. Phylogenetic analysis of its small subunit ribosomal DNA sequences demonstrated that the parasite belonged to the *Nucleospora* group clade. The histological, ultrastructural, and molecular data support the emergence of a new species in the genus *Nucleospora*. This is the first report of *Nucleospora* species in Asia and threatened syngnathid fishes.

## Introduction

Seahorses are highly modified pipefish and are commonly traded for traditional medicine, ornamental display, and aquarium fish (Koning and Hoeksema, 2021). As a threatened fish species, seahorses are being commercially cultured to solve overexploitation and the increasing demand in global trade. The high-density pressure in the culture makes them more vulnerable to many infectious diseases, including vibriosis, mycobacteriosis, fusariumsis, parasitosis, and microsporidiosis (Koldewey and Martin-Smith, 2010; LePage et al., 2015). Few microsporidians have been reported in the seahorse. Only *Glugea heraldi* has been formally described by Blasiola Jr. (1979) as infecting the wild-caught *Hippocampus erectus* (Teleostei: Syngnathidae).

Microsporidia are obligate intracellular eukaryotic parasites with a close fungal relationship in evolutionary origin (Corsaro et al., 2016; Bass et al., 2018). Most microsporidian species live in the cytoplasm of their host cells directly or indirectly, but few microsporidians exhibit unique intranuclear infections, such as *Enterospora* spp. (Stentiford et al., 2007; Palenzuela et al., 2014; Yan et al., 2018), *Nucleospora* spp. (Chilmonczyk et al., 1991; Freeman and Kristmundsson, 2013), and *Desmozoon lepeophtherii* (synonym *Paranucleospora theridion*) (Freeman and Sommerville, 2009; Freeman and Sommerville, 2011). To date, no intranuclear microsporidians have been described as infecting syngnathid fishes.

In March 2019, a disease of the cultured lined seahorse *Hippocampus erectus* broke out in Hainan province, China, characterized by a severe intestinal lesion. By microscopic examination, numerous microsporidian spores were discovered in the intestinal mucus and white feces of diseased fishes. In this paper, we applied both ultrastructural observation and molecular analyses to identify the microsporidian found in the *Hippocampus erectus* intestine. The morphological, ultrastructural, and molecular data support the idea that it is a new intranuclear species of the genus *Nucleospora*.

## Materials and Methods

### Sample Collection

Sick seahorses, *Hippocampus erectus*, were collected from recirculating tank systems in the Qionghai Research Center of the East China Sea Fisheries Research Institute, Hainan province, China (19°22′24″N, 110°40′10″E) in March and August of 2019. The temperature of the water ranged from 18°C to 28°C, and the salinity ranged from 28 to 30 ppt. A total of 153 samples (body length: 2~10 cm) were necropsied after anesthetization with MS222 for euthanasia. All fish experiments were conducted according to the national standard guidelines for ethical review of animal welfare (GB/T 35892-2018).

### Histology

The gastrointestinal tracts were partially cut along the longitudinal axis. The inner gastrointestinal contents and some epithelial cells were scraped off, smeared on microscopic slides, and observed under a Leica DM4B light microscope. After light microscopic examination, the tissues containing microsporidian spores were preserved in different fixative solutions for processing for histology, electron microscopy, and molecular biology analyses, respectively.

Portions of the gills, body musculature, heart, kidney, liver, spleen, intestine, and gall bladders were fixed in Davidson’s fixative for 24 h. The tissues were then processed using routine histological techniques. Tissue sections (5 μm) were stained using Masson’s trichrome method (Chang et al., 2019). Images were captured using a Leica camera DMC6200 with the Leica Application Suite X (version 3.4.2) software.

### Transmission Electron Microscopy

Small pieces of affected intestine tissues were fixed in 2.5% glutaraldehyde in 0.1 M phosphate buffer (pH 7.4) at 4°C for 24 h. The fixed tissues were rinsed three times in 0.1 M phosphate buffer and then postfixed in 1% osmium tetroxide (OsO_4_) for 1 h at 4°C. After washing three times with the same buffer, specimens were dehydrated through a graded acetone series, embedded in epoxy resin 812, and then polymerized at 60°C for 16 h. Semithin sections (1 μm) were stained with Toluidine Blue to target infected areas. Ultrathin sections (60–80 nm) were mounted on uncoated copper grids and stained with uranyl acetate and lead citrate. Samples were examined using a Tecnai G2 Spirit Biotwin transmission electron microscope at 80 kV.

### Polymerase Chain Reaction Amplification

The counterpart tissues were preserved in 100% ethanol at the time of dissection. After ethanol evaporation, total genomic DNA was extracted from three fish using the TIANamp Marine Animals DNA Kit (Tiangen, China), following the manufacturer’s instructions. Isolated DNA was eluted in 50 μl of TE buffer and stored at −20°C for polymerase chain reactions (PCR).

The previously published primer set, 18F (5′-CACCAGGTTGATTCTGCC-3′) and 580R (5′-GGTCCGTGTTTCAAGACGG) (Vossbrinck et al., 1993; Vossbrinck et al., 2004), was used to amplify the microsporidian rDNA sequence, including the whole small subunit rRNA gene (SSU rRNA), the internal transcribed spacer (ITS), and the partial large subunit rRNA gene (LSU rRNA).

Amplifications were performed in a total volume of 50 μl containing 25 μl of PrimeSTAR^®^ Max premix (Takara, China), 2 μl of 10 μM for each pair of primers, 19 μl of sterile water, and 2 μl of genomic DNA template. PCR conditions were 95°C for 5 min, followed by 35 cycles of 94°C for 45 s, 52°C for 35 s, and 72°C for 90 s, with a final extension at 72°C for 5 min.

### Cloning and Sequencing Analysis

The amplified PCR products were detected by 1% agarose gel electrophoresis and purified using an Agarose Gel DNA Purification Kit (Takara, China). The purified fragments were cloned into the pMD19 T-vector, transformed into competent *Escherichia coli* DH5α cells, and plated on Luria–Bertani agar plates containing ampicillin (Amp-LB). After overnight culture, the positive clones verified by colony PCR were amplified in liquid Amp-LB medium. PCR fragments from bacterial clones containing the correct insert size were sequenced with primers M13F/M13R using an Applied Biosystems 3730xl DNA analyzer by Sangon Biotech (Shanghai) company. The internal primers 870F (5′-TGCGGCTTAATTTGACTCAAC-3′) and 870R (5′-GTTGAGTCAAATTAAGCCGCA-3′) were used for the complete overlap in DNA sequencing (Freeman et al., 2013). The obtained sequences were carefully examined and assembled using BioEdit software (version 7.2.5).

### Phylogenetic Analysis

The sequences were evaluated using the BLASTn programs in NCBI to compare them with those available in the microsporidian SSU rDNA databases. To assess the phylogenetic position of this species with other microsporidians, 28 microsporidian SSU rDNA sequences were selected from the NCBI GenBank database based on the BLAST search results and literatures. Sequences were aligned using the ClustalW algorithm in MEGA software version 10.0 with default settings (Kumar et al., 2018). The model of “General Time Reversible Model + gamma-distributed (GTR+G)” was chosen based on estimated results of “Find Best DNA/Protein Models (ML)” to construct the Maximum likelihood (ML) tree in MEGA X. The robustness of the resulting tree was assessed using 1,000 bootstrap replicates. The Bayesian phylogenetic inference was performed using MrBayes version 3.2.7 (Ronquist et al., 2012). The GTR substitution model with gamma-distributed rate variation across sites was set for tree reconstruction. Bayesian searches were run for 1,000,000 generations and sampled every 1,000 generations. The convergence of Markov chain Monte Carlo (MCMC) analyses was diagnosed in the Tracer version 1.7.2.

## Results

### Clinical Signs and Epidemiology

Infected lined seahorses exhibited anorexia, spiritlessness, occasional white feces, and chronic mortality. After necropsy, there were no obvious tissue damage and other infection symptoms. The gut of the severely infected fishes had a slight whitish appearance. No other organ observed was found infected. Microsporidian infections were found in both juvenile and adult stages of the lined seahorses.

### Light Microscopy

**Figure 1:**
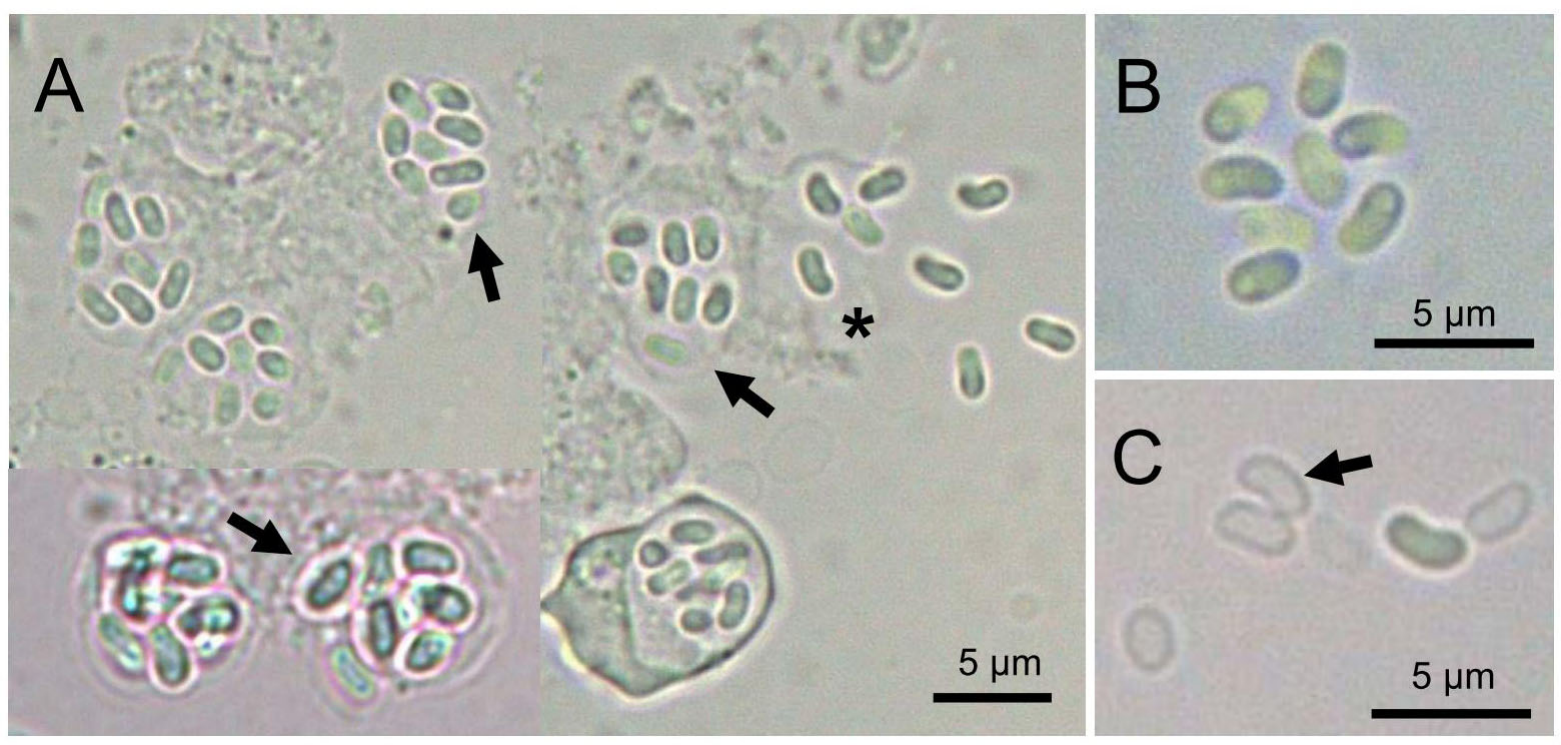
Light microscopy of *Nucleospora hippocampi* n. sp. **(A)** The infected enterocytes contain eight spores (arrows). Eight spores are released from the ruptured enterocyte (asterisk). **(B)** Higher magnification of eight fresh spores. **(C)** The arrow shows the empty spore shell.

Microscopic examination revealed that microsporidian spores were commonly present in the intestinal tract and feces of infected *Hippocampus erectus*. In a wet mount, the infected host enterocyte cell generally contained eight spores (**Figure 1A**). The spores had an ellipsoid shape (**Figure 1B**). Occasionally, empty spores were seen in the feces (**Figure 1C**). Fresh spores averaged (2.66 ± 0.21) × (1.44 ± 0.19) μm [*n* = 50; range (2.04–3.12) × (0.99–1.85) μm]. The prevalence of microsporidian infection among individuals was 22.2% (34 of 153).

### Histopathology

**Figure 2:**
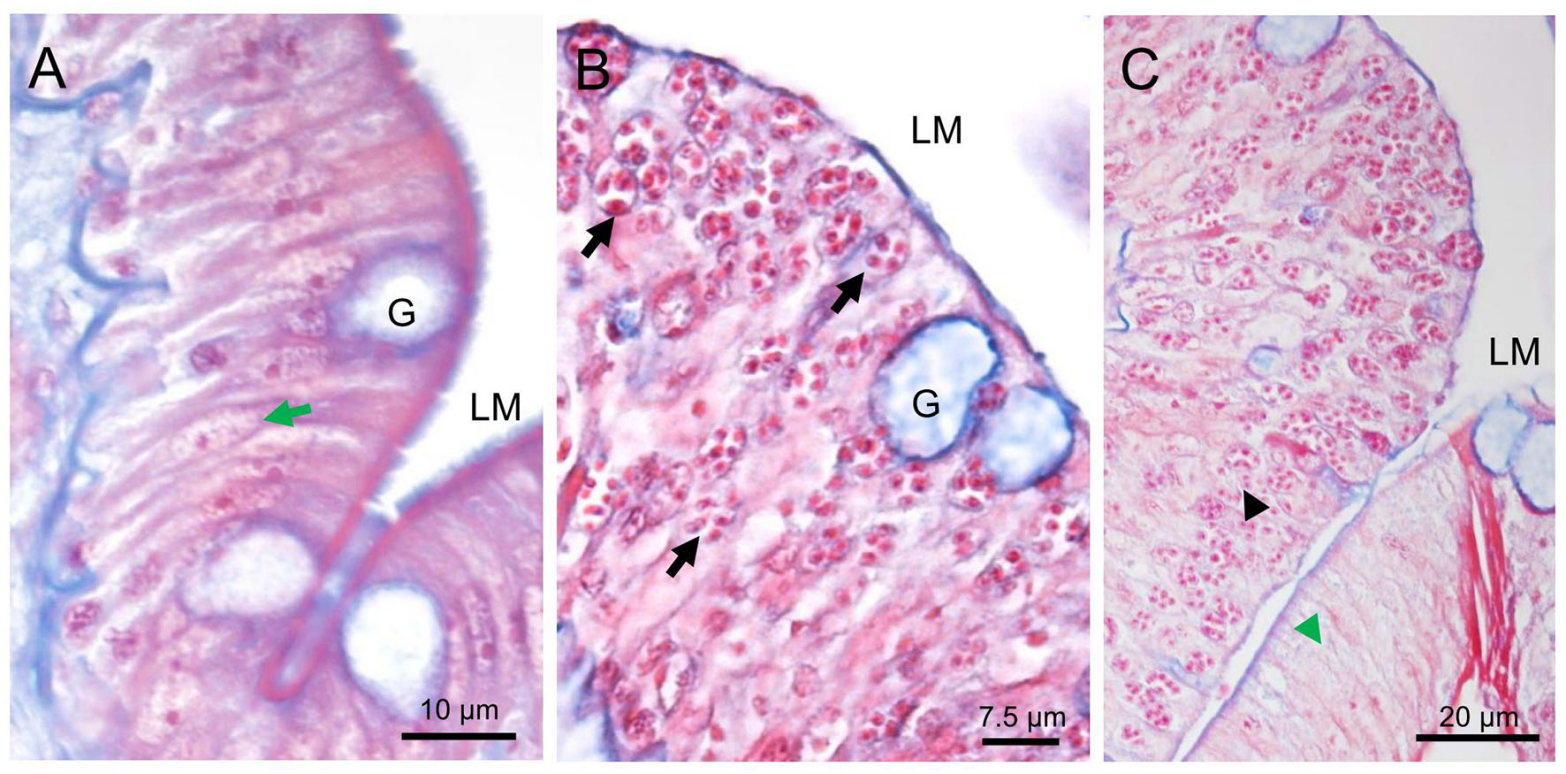
Histopathology of *Nucleospora hippocampi* n. sp. infection in the intestinal epithelia of the *Hippocampus erectus*. Masson’s stain. **(A)** Normal columnar epithelium of the intestine: intestinal lumen (LM), host nucleus (green arrow), and goblet cell **(G)**. **(B)** The intestinal epithelium with microsporidian infection (black arrows). **(C)** The infected region (black arrowhead) and uninfected region (green arrowhead) of the intestinal epithelium layer.

**Figure 3:**
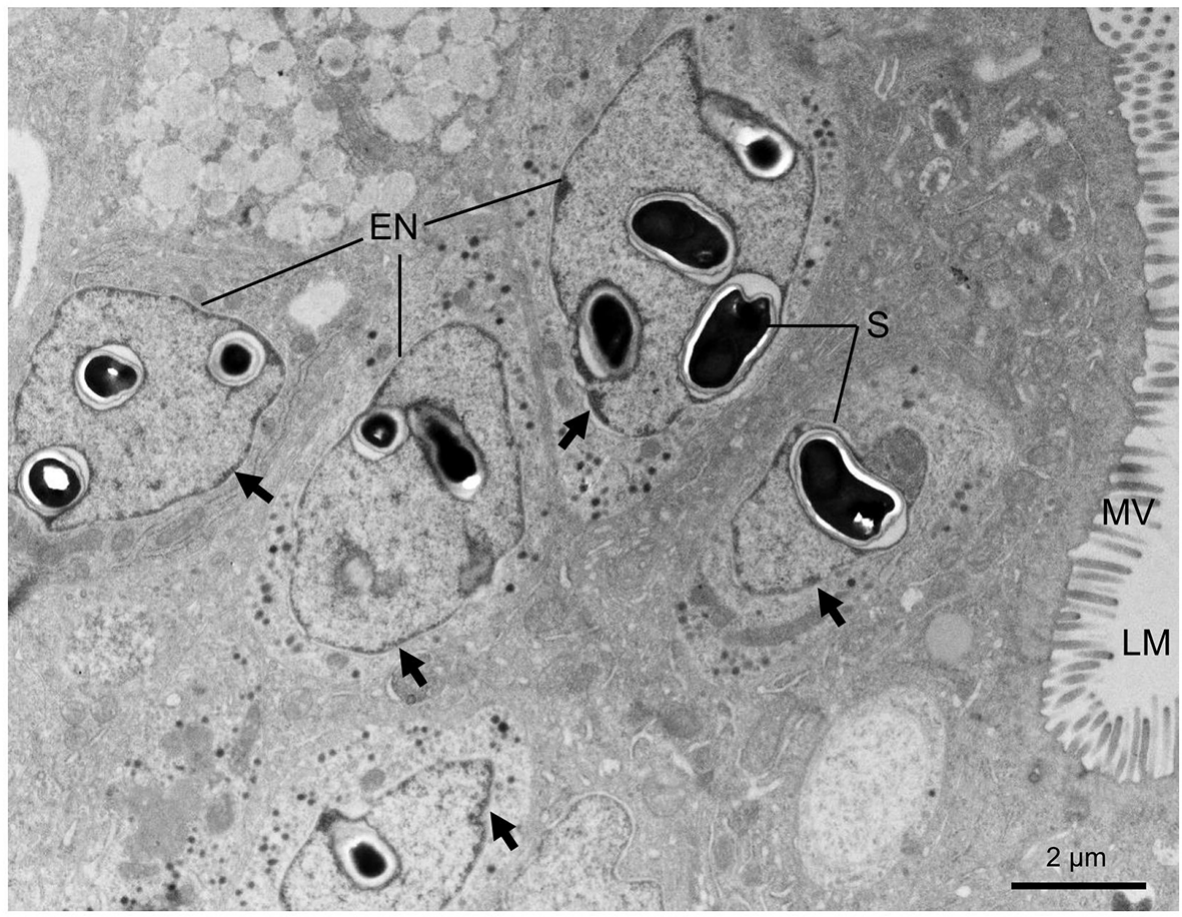
Transmission electron micrographs showing the microsporidian *Nucleospora hippocampi* n. sp. infecting the intestine cells of the *Hippocampus erectus*. Intranuclear infections occur in five enterocytes. Affected nuclei with marginalized chromatin (black arrows). LM, intestinal lumen; MV, microvilli; S, spore; EN, enterocyte nucleus.

The varying numbers of cells in the epithelium layer were infected compared with the normal intestinal tract tissue (**Figures 2A, B** **)**. Affected cells include the enterocytes and goblet cells (**Figures 2B**). Based on the overwhelming infection observed, the former were the preferred target cells for the parasite. Parasite stages appeared as eosinophilic granular and red staining (**Figures 2B, C** **)**. The microsporidians were seemingly surrounded by the membrane structures (**Figures 2B, C** **)**, which were subsequently confirmed to be the host’s nuclear envelope by transmission electron microscopy (TEM) (**Figure 3**). No infection was observed in other host organs.

### Ultrastructure of Microsporidian Development

**Figure 4:**
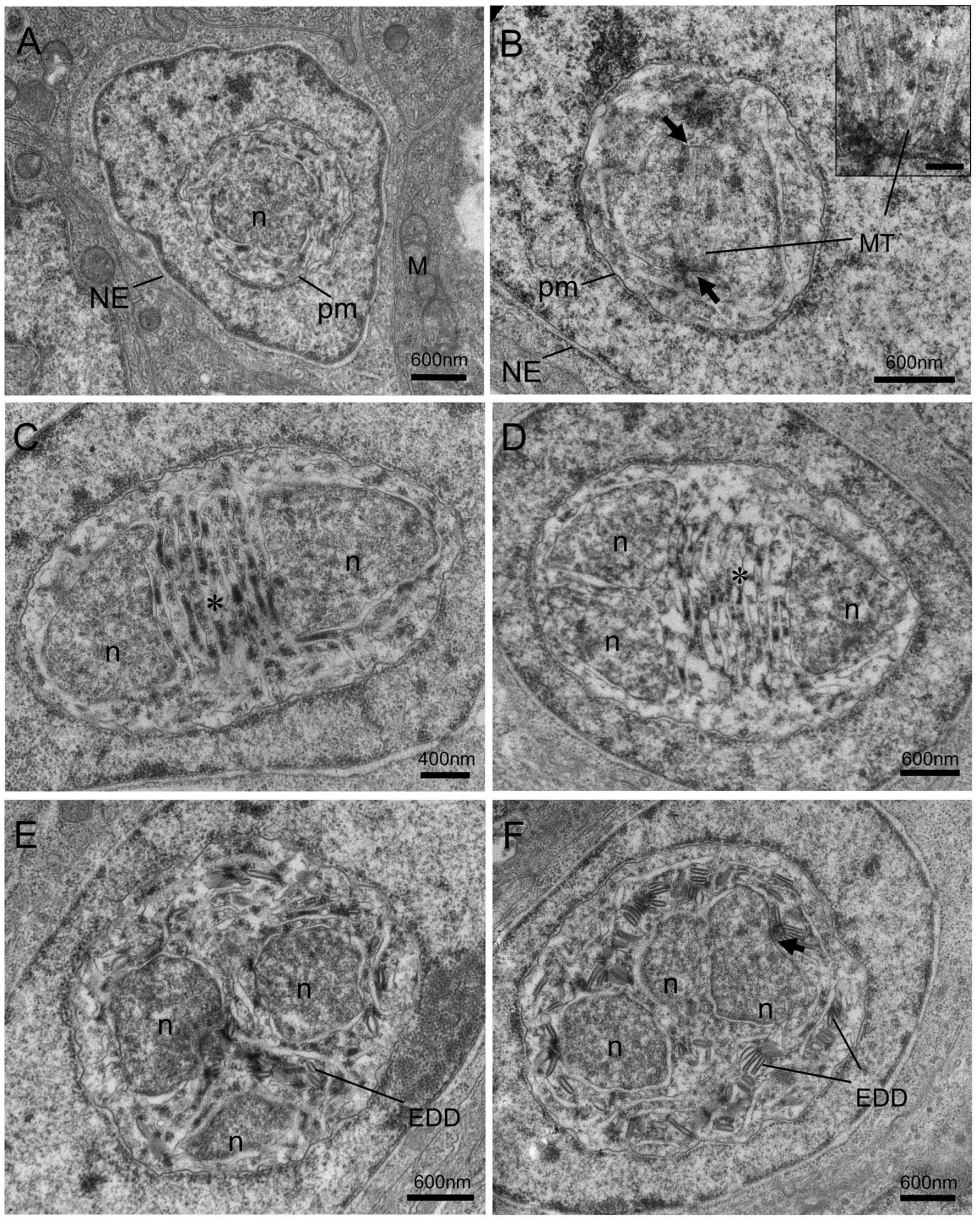
Transmission electron micrographs of the merogonic and early sporogonic stages of *Nucleospora hippocampi* n. sp. in the nuclei of intestinal epithelial cells of the *Hippocampus erectus*. **(A)** Early uninucleate meront stage within the nucleoplasm of the host nucleus. Host nuclear envelope (NE), host mitochondria (M), the monokaryon (n), and plasma membrane (pm) of the meront. **(B)** The uninucleate meront undergoing karyokinesis. Long microtubules (MT) radiate from two spindle plaques (black arrows). Inset showing the details of the microtubules and spindle plaque. Insert scale bar = 0.1 μm. **(C)** The binucleate meront contains two nuclei (n) separated by claviform cytoplasm (asterisk). **(D)** The trinucleate meront with three nuclei and claviform cytoplasm (asterisk). **(E)** The electron-dense discs (EDD) appear in the cytoplasm of late trinucleate meront (or early sporogonial plasmodium). **(F)** An early sporogonial plasmodium with increasing EDD around three nuclei. The spindle plaque (black arrow) forming on one nucleus indicates nucleus division again.

**Figure 5:**
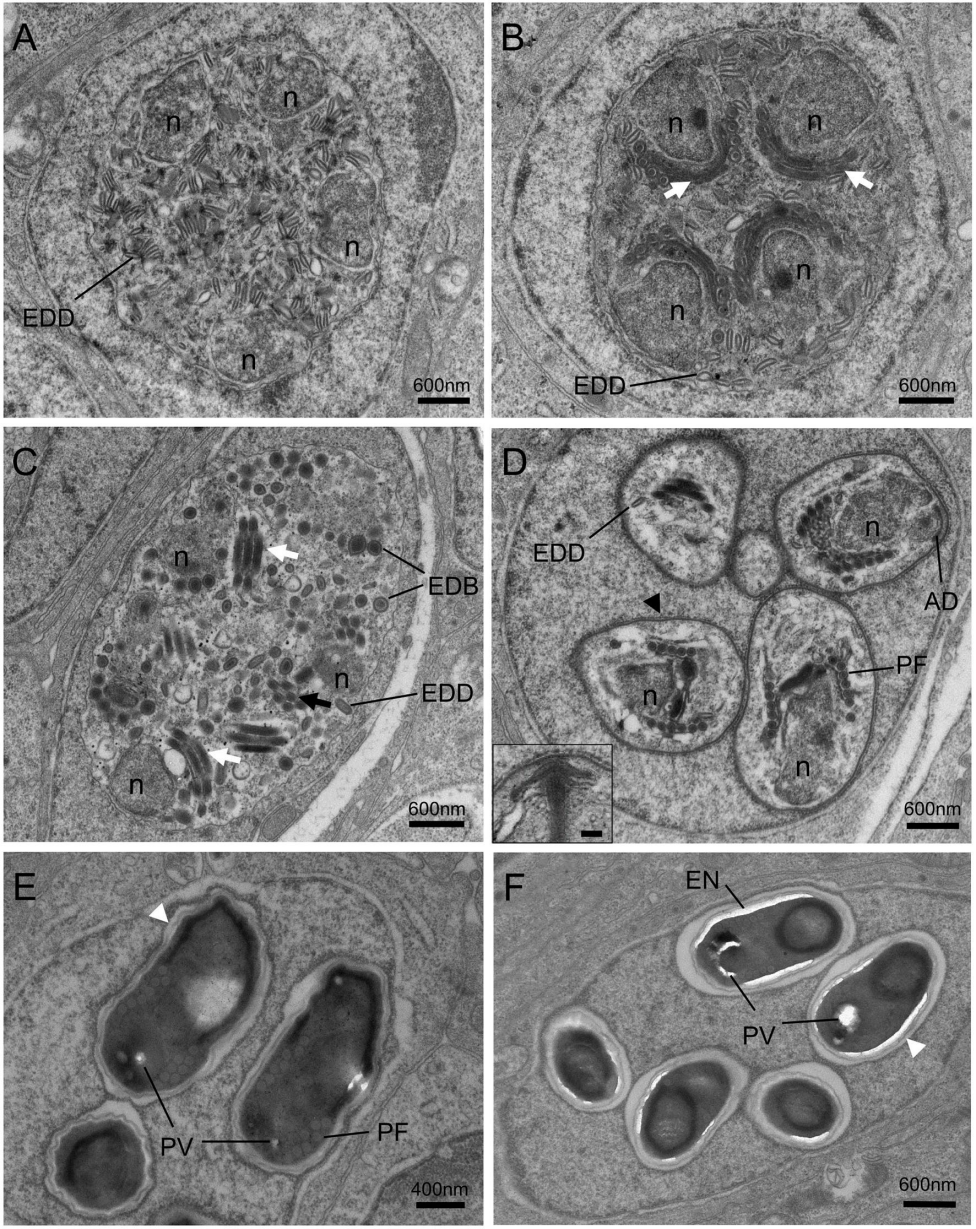
Transmission electron micrographs of the sporogonic stages of *Nucleospora hippocampi* n. sp. in nuclei of intestinal epithelial cells of *Hippocampus erectus*. **(A)** The sporogonial plasmodium contains numerous electron-dense discs (EDD) and four nuclei (n) distributed close to the inner edge of the cytomembrane. **(B)** Multinucleate sporogonial plasmodium contains four sets of the primordial polar tube (white arrows). EDD is associated with primordial polar tube formation. **(C)** Late-stage sporogonial plasmodium with increased precursors of the polar tube (white arrows) and reduced EDD. The EDD transform into rounded electron-dense bodies (EDB), then the EDB coalesce into primordial turns of the polar tube (black arrow). **(D)** The sporoblasts (five are visible) develop in direct contact with the host nucleoplasm. The sporoblasts showing parasite nucleus (n), coiled polar tube with 6–7 turns (PF), and thickened plasmalemma (arrowhead). Insert showing the magnification of anchoring disk (AD). Insert scale bar = 0.1 μm. **(E)** Late immature spore with preformed winding spore wall (white arrowhead), posterior vacuole (PV), and polar tube (PF). **(F)** Mature spore with smooth spore wall (white arrowhead) and thickening electron lucent endospore (EN).

Different proliferative stages of the parasite directly develop in the nucleoplasm of enterocytes. The earliest stage observed was rounded uninucleate meronts, 1.3~2.0 μm in diameter, characterized by a large single nucleus and a simple plasmalemma (**Figure 4A**). The outer layer of the meront plasmalemma deposited electron-dense materials (**Figures 4A, D** **)**. The uninucleate meront underwent mitosis to form binucleate meront (**Figures 4B, C** **)**. During mitosis, the spindle plaques and microtubules appeared; the meront nuclear envelope preserved; the claviform cytoplasm, which might be endoplasmic reticulum, was increased. The binucleate meront continued to divide without cytokinesis, giving rise to a multinucleate meront with numerous claviform cytoplasm (**Figure 4D**). The transformation from multinucleate meront to multinucleate sporogonial plasmodium was easily recognized by the formation of the electron-dense disc (EDD) structures (**Figure 4E**). As development progressed, more electron-dense discs were synthesized in the early sporogonial plasmodium cytoplasm. The nuclei underwent further division (**Figure 4F**) and migrated to the inner edge of its cytomembrane (**Figures 5A, B** **)**.

The electron-dense discs of multinucleated sporogonial plasmodium gradually decreased and turned into rounded electron-dense bodies (EDB) (**Figures 5A–C**), which were the raw materials for the manufacture of the primordial polar tube. At this stage, several units of polar tube precursors, each closing to the individual nuclei respectively, were assembled in the late sporogonial plasmodium (**Figures 5B, C** **)**. The sporogonial plasmodium was separated into several uninucleate sporoblasts by cytokinesis. Individual sporoblasts could not divide further. During the development, the precursor of an anchoring disk formed in each sporoblast (**Figure 5D**), and the number of regularly arranged polar filament coils increased at this developing stage. Maturation of liberated sporoblasts into immature spores was characterized by the transformation of a moderately thickened membrane into a winding spore wall (**Figure 5E**). The electron-lucent endospore became visible and progressively thicker as the spore matured (**Figures 5E, F** **)**. Considering that the host nucleus was spherical and the ultrathin section only showed a cut surface of the host nucleus, we hypothesized that eight sporoblasts were generated from the multinucleated sporogonial plasmodium (**Figure 5D**) and that these eight sporoblasts eventually developed into eight spores in one host nucleus.

**Figure 6:**
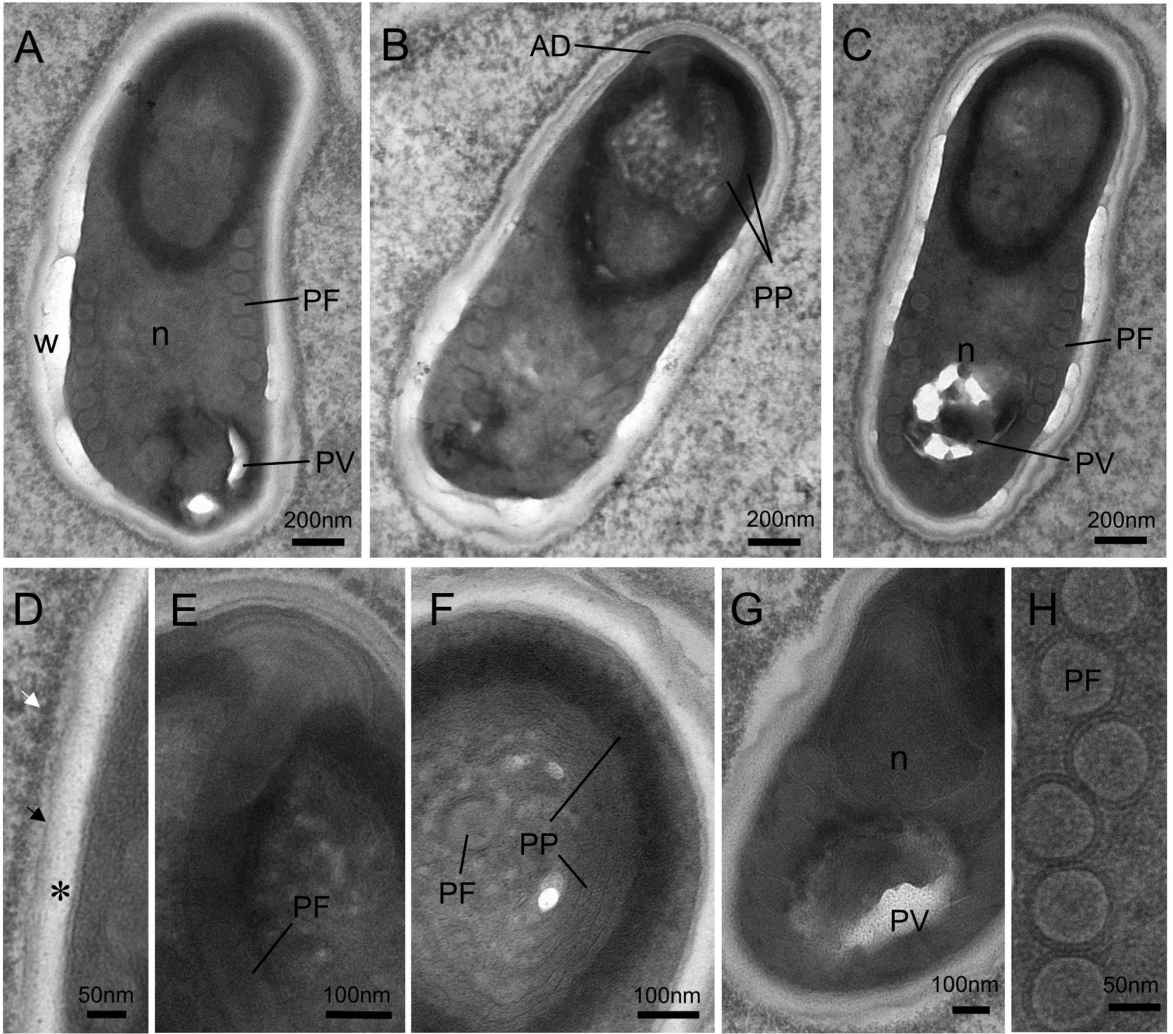
Transmission electron micrographs showing the ultrastructure of mature spores of *Nucleospora hippocampi* n. sp. in the *Hippocampus erectus*. **(A–C)** Three spores showing the typical microsporidian structures and organelles. The spore wall (W), anchoring disk (AD), polaroplast (PP), posterior vacuole (PV), polar filament (PF), and the single nucleus (n) are indicated. **(D)** Ultrastructural detail of spore wall showing the exospore (black arrow), the endospore (asterisk), and the plasmalemma (arrowhead). Notice the electron-dense materials deposited on the outer surface of the exospore (white arrow). **(E)** Detail of terminal anchoring disk and associated polar tube (PF). **(F)** High magnification of a transverse section of a spore showing the lamellar region of the polaroplast (PP). **(G)** The single nucleus (n) is situated beside the posterior vacuole (PV). **(H)** Detail of the isofilar polar filament (PF).

Mature spores were ellipsoidal and slightly bent (**Figure 6A**). The spores were measured at 1.93 ± 0.17 (1.68–2.49; *n* = 50) μm in length and 0.97 ± 0.08 (0.74–1.24, *n* = 50) μm in width. The spore wall was composed of a thick electron-transparent endospore and an electron-dense exospore. A layer of electron-dense materials was deposited outside the spore wall (**Figure 6D**). The thickness of the spore wall was about 70 nm. Inside the spores, the anchoring disc was umbrella-shaped (**Figure 6E**); the polaroplast consisted of two distinct lamellar parts: a tightly packed lamellae part and a loose tubular internal part composed of 5 layers (**Figures 6B, F** **)**; the single nucleus was located in the midregion of the spore (**Figure 6G**); the posterior vacuole was observed at the posterior end of spore (**Figures 6A, C, G** **)**; and the isofilar polar tube usually formed 7-8 coils in a single layer close to the spore wall, with a diameter of approximately 80 nm (**Figure 6H**).

### Molecular Analyses

The PCR fragments amplified using the primer pair 18F/580R were 1,922 bp in length, containing complete SSU rDNA (1,269 bp), a complete ITS region (269 bp), and partial LSU rDNA (384 bp). Three rDNA sequences of *Nucleospora hippocampi* n. sp. isolated from three infected *Hippocampus erectus* were deposited in GenBank with accession numbers MW229242 to MW229244, respectively. These rDNA sequences showed low intraspecific variations (>99.4% similarity).

The BLASTn search results revealed no known microsporidian sequences available in GenBank that matched exactly to the obtained sequences. Comparison of the nucleotide sequences showed that the *Nucleospora hippocampi* n. sp. SSU rDNA data (1,269 bp of Genbank MW229243) showed a 96% sequence identity (1,202/1,251 bp) to *Nucleospora salmonis* (Genbank U78176), a 95% sequence identity (1,200/1,263 bp) to *Nucleospora cyclopteri* (Genbank KC203457), and a 96% sequence identity (391/406 bp) to *Nucleospora braziliensis* (Genbank KT777455). The SSU rDNA sequences of other microsporidians, including *Obruspora papernae* (Genbank HG005137), *Paranucleospora theridion* (Genbank KR187185), *Enterocytozoon hepatopenaei* (Genbank KX981865), *Enterospora canceri* (Genbank HE584634), and *Enterocytozoon bieneusi* (Genbank L07123), showed 90%, 89%, 87%, 86%, and 83% identity with *Nucleospora hippocampi* n. sp., respectively.

**Figure 7:**
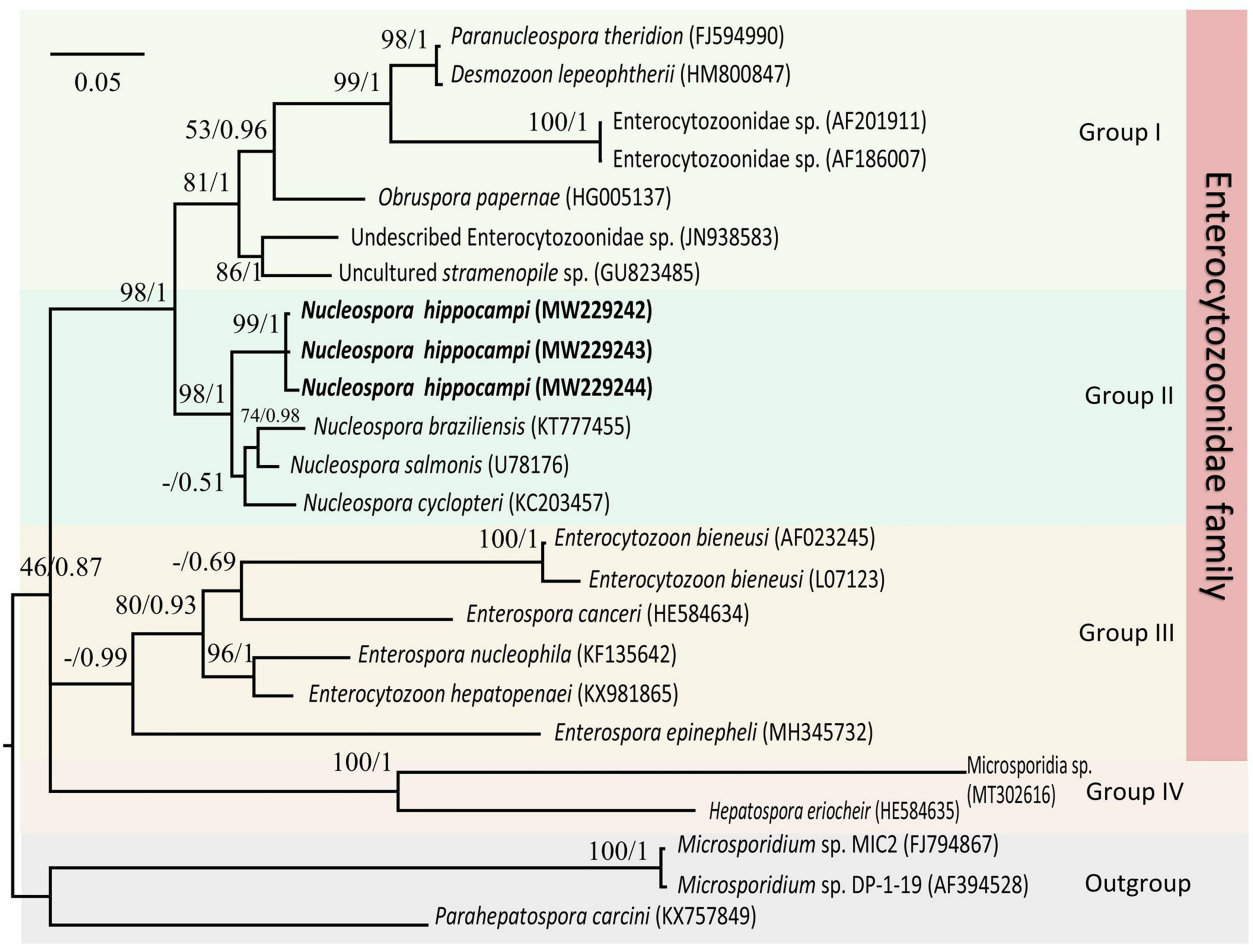
The Maximum likelihood and Bayesian analyses based on SSU rDNA sequences showing the relationship of *Nucleospora hippocampi* n. sp. and other selected microsporidian species. *Parahepatospora carcini* (KX757849), *Microsporidium* sp. MIC2 (FJ794867), and *Microsporidium* sp. DP-1-19 (AF394528) are used as the out-group. Values at nodes represent Maximum-likelihood bootstrap support percentages (BP)/Bayesian posterior probabilities (PP). The scale bar represents the estimated number of substitutions per nucleotide site.

The Maximum likelihood and Bayesian phylogenetic analyses produced similar tree topology (**Figure 7**), placing three *Nucleospora hippocampi* n. sp. isolates within a highly supported clade (bootstrap percentages (BP) = 99; posterior probabilities values (PP) = 1). Furthermore, the new microsporidian was strongly supported to cluster with *Nucleospora salmonis*, *Nucleospora cyclopteri*, and *Nucleospora braziliensis* in the large *Nucleospora* clade (BP = 98; PP = 1). *Nucleospora* spp. was classified in group II as a sister taxon to group I, which included *Desmozoon* (=*Paranucleospora*), *Obruspora*, and several undescribed genera. The family Enterocytozoonidae, including the genera: *Enterocytozoon*, *Enterospora*, *Nucleospora*, *Obruspora*, and *Desmozoon*, was closely related to the family Hepatosporidae (group IV) (Stentiford et al., 2011). Based on the new classification view of Microsporidia (Vossbrinck and Debrunner-Vossbrinck, 2005), this parasite belonged to clade IV, known as class Terresporidia.

### Taxonomic Description

#### Taxonomic rankings

Super-group: Opisthokonta Cavalier-Smith (1987)

Super-phylum: Opisthosporidia Karpov et al. (2014)

Phylum: Microsporidia Balbiani (1882)

Class: Terresporidia Vossbrinck and Debrunner-Vossbrinck (2005)

Family: Enterocytozoonidae Cali and Owen (1990)

Genus: *Nucleospora* Hedrick, Groff and Baxa (1991)

#### *Nucleospora hippocampi* n. sp.

**Figure 8:**
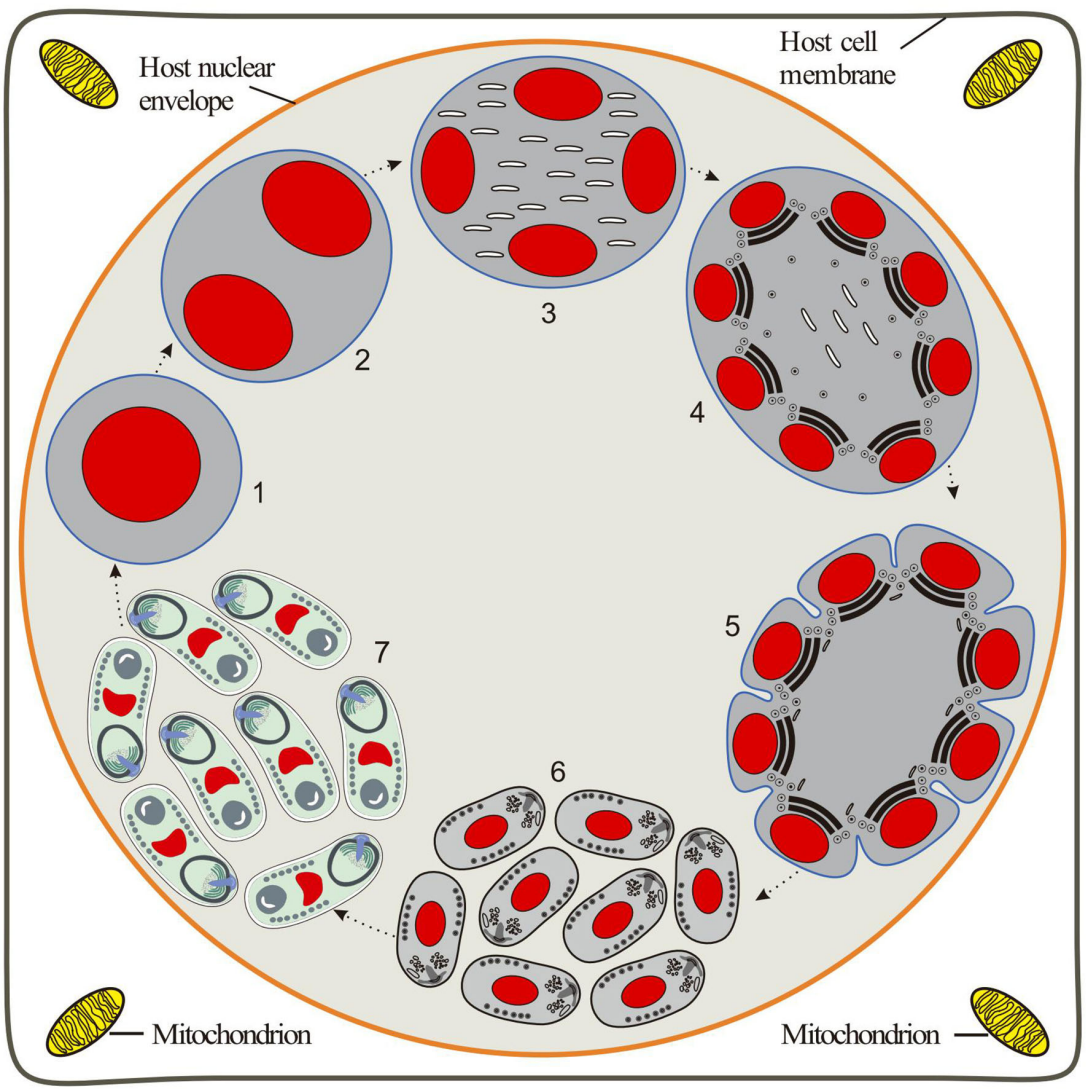
Diagrammatic representation of the proposed intranuclear life cycle of *Nucleospora hippocampi* n. sp. (1) Uninucleate meront free in the host nucleus. (2) Binucleate meront formed by nucleus mitosis without cytokinesis. (3) The nuclear divides again to produce a tetra-nucleate meront with numerous claviform cytoplasm. (4) The proliferative sporogonial plasmodium shows electron-dense disks and eight units of polar tube precursors around the inner edge of its plasmalemma. (5) The syncytial sporogonial plasmodium produces eight uninucleate sporoblasts by rosette-like budding. The invagination of plasmalemma segregates each nucleus with polar tube precursors into each sporoblast. (6) The individual sporoblasts develop into immature spores. (7) Eight mature spores are formed in direct contact with the host nucleoplasm.

Diagnosis of the species: Monokaryotic spores are elongated ovoids that are slightly bent. Eight spores are frequently observed in one host nucleus without forming the interfacial membrane. Fresh spores measure approximately 2.66 × 1.44 μm. Fixed spores are 1.93 × 0.97 μm in size. The spore wall is about 70 nm of thickness. The single nucleus is positioned in the posterior half of the spore. The isofilar polar tube coils have 7–8 turns arranged in a single layer, about 80 nm in diameter. The posterior vacuole is situated at the posterior end of the spore and is surrounded by the polar filaments. Polaroplast with two regions: densely arranged lamellae and loose tubular lamellae. The intranuclear life cycle of *Nucleospora hippocampi* n. sp. is inferred in **Figure 8**.

Type-host: Lined seahorse *Hippocampus erectus* (Teleostei: Syngnathidae).

Type-locality: The Hainan province, China (19°22′24″N, 110°40′10″E).

Site of infection: Intestinal epithelial cells.

Prevalence: 34 of 153 (22.2%) lined seahorses examined by light microscopy.

Type-material: Paraffin sections and resin-embedded tissues are deposited in the East China Sea Fisheries Research Institute, China Academy of Fishery Sciences, Shanghai.

Type SSU rDNA sequence: GenBank accession No. MW229243.

Etymology: The specific epithet “hippocampi” derives from the generic name of the host.

## Discussion

### *Nucleospora hippocampi* n. sp. Is a New Microsporidian

The ultrastructural morphology of this microsporidian development shows one of the key features of the family Enterocytozoonidae: the spore organelles are almost fully developed before the sporogony plasmodium divides into sporoblasts (Sprague et al., 1992). Moreover, molecular data further reveals its phylogenetic position within the *Nucleospora* group on the Enterocytozoonidae family.

To date, four *Nucleospora* species have been described: *Nucleospora salmonis* (previously called *Enterocytozoon salmonis*), *Nucleospora secunda*, *Nucleospora cyclopteri*, and *Nucleospora braziliensis*. The biological features of all members are compared in **Table 1**. The presented *Nucleospora hippocampi* n. sp. is distinguished from other *Nucleospora* species by differences in shape and size, the number of turns of the polar tube, host cell type and tissue tropism, and the number of spores in a host cell section. It is worth mentioning that the SSU rDNA sequence divergences between *Nucleospora hippocampi* n. sp. and other *Nucleospora* species also support the morphological differences. Taken together, a new species of the *Nucleospora* genus is proposed for establishment. It is the first report of *Nucleospora* species in the seahorse.

**Table 1:** Biological and molecular data of the microsporidians in the genus *Nucleospora*.

Species	Spore shape	Spore size (μm)	No. of spores in per nucleoplasm	PF coils	Infection cell type	Tissue tropism	Host	Habitat	SSR DNA (GenBank No.)	References
*Nucleospora salmonis*	Ovoid	2 × 1 (TEM)	1–8	8–12	Hematopoietic cell, blood leukocyte	Spleen, kidney	*Oncorhynchus tshawytscha*	Euryhaline	U78176	Chilmonczyk et al. (1991); Docker et al. (1997)
*Nucleospora secunda*	Ellipsoid	1.65 × 0.82 (TEM)	7–18	4–5	Enterocyte	Intestine	*Nothobranchius rubripinnis*	Freshwater	Not available (na)	Lom and Dyková (2002)
*Nucleospora braziliensis*	Ovoid	1.34 × 0.61 (fresh)	na	na	na	Gill, gut, heart, kidney, liver, muscle, spleen, and stomach	*Oreochromis niloticus*	Euryhaline	MW491352	Rodrigues et al. (2017)
*Nucleospora cyclopteri*	Elongate ovoid	2.53 × 1.04 (TEM), 3.12 × 1.30 (fresh)	1–14	10–12	Lymphocyte	Kidney	*Cyclopterus lumpus*	Marine	KC203457	Freeman et al. (2013); Freeman and Kristmundsson (2013)
*Nucleospora hippocampi* n. sp.	Elongate ovoid, slightly bent	2.66 × 1.44 (fresh), 1.93 × 0.97 (TEM)	1–8	7–8	Enterocyte	Intestine	*Hippocampus erectus*	Marine	MW229243	This study

### Awareness of the Potential Threat of *Nucleospora hippocampi* n. sp. to the Seahorse

This parasite belongs to the emergent *Enterocytozoon* group Microsporidia (EGM) (Stentiford et al., 2019). EGM has been responsible for many emergent diseases in varied hosts over the past 50 years. For instance, *Enterocytozoon bieneusi* is the most prevalent human microsporidian, especially infecting the intestinal epithelial cells of severely immune-suppressed humans, and is associated with self-limiting diarrhea, malabsorption, and wasting (Matos et al., 2012). *Enterocytozoon hepatopenaei* has recently caused substantial economic losses in global shrimp aquaculture and is associated with slow growth and white-face syndrome (Chaijarasphong et al., 2020). *Enterospora nucleophila* infects the farmed gilthead sea bream (*Sparus aurata*), causing an emaciation syndrome and significant mortality (Palenzuela et al., 2014). In particular, other species in the *Nucleospora* genus, such as *Nucleospora salmonis* and *Nucleospora cyclopteri*, have caused economic damage in fish aquaculture (Sakai et al., 2009; Naung et al., 2021). Hence, as one member of this highly pathogenic EGM, the potential threat of *Nucleospora hippocampi* n. sp. should be taken seriously in cultured seahorse.

For many years, the theory of host–parasite population dynamics has long held the interest of ecologists. In some hosts, the role of microsporidia in population regulation has been reported (Kohler and Hoiland, 2001; Stentiford et al., 2013), such as daphnia, locust, honeybee, and mosquito (Han et al., 2020). Seahorses are fascinating sea creatures, but at present, the global seahorse populations are in severe decline. Furthermore, all seahorse species are on the International Union for Conservation of Nature (IUCN) Red List. Because infection data for wild seahorses are not available, it is unclear whether *Nucleospora hippocampi* n. sp. plays a role in exacerbating the decline of seahorse resources. Further research on epidemiology is needed to evaluate its effect on host richness.

## Data Availability Statement

The datasets presented in this study can be found in online repositories. The names of the repository/repositories and accession number(s) can be found below: https://www.ncbi.nlm.nih.gov/genbank/, MW229242.

## Ethics Statement

The animal study was reviewed and approved by East China Sea Fisheries Research Institute. All fish experiments were carried out in accordance with the laboratory animal guidelines for ethical review of animal welfare (China national standard GB/T 35892-2018).

## Author Contributions

WF and HT designed the experiments. YW performed the experiments and prepared the manuscript. NY, YH, and JZ participated in pathology experiment. LL constructed the figures. XZ and XL assisted in sample collection. SZ, RM, and XCL assisted in molecular analyses. All authors revised and approved the submitted version of the manuscript.

## Funding

This work was supported by the Central-Level Non-profit Scientific Research Institutes Special Funds (East China Sea Fisheries Research Institute) (No. 2019M03) and the Central Public-interest Scientific Institution Basal Research Fund (Chinese Academy of Fishery Sciences) (NO. 2020TD41).

## Conflict of Interest

The authors declare that the research was conducted in the absence of any commercial or financial relationships that could be construed as a potential conflict of interest.

## Publisher’s Note

All claims expressed in this article are solely those of the authors and do not necessarily represent those of their affiliated organizations, or those of the publisher, the editors and the reviewers. Any product that may be evaluated in this article, or claim that may be made by its manufacturer, is not guaranteed or endorsed by the publisher.
